# Acute exercise in a hot environment increases heat shock protein 70 and peroxisome proliferator-activated receptor γ coactivator 1α mRNA in Thoroughbred horse skeletal muscle

**DOI:** 10.3389/fvets.2023.1230212

**Published:** 2023-08-21

**Authors:** Yusaku Ebisuda, Kazutaka Mukai, Yuji Takahashi, Toshinobu Yoshida, Aoto Kawano, Tsubasa Matsuhashi, Hirofumi Miyata, Masayoshi Kuwahara, Hajime Ohmura

**Affiliations:** ^1^Sports Science Division, Equine Research Institute, Japan Racing Association, Shimotsuke, Japan; ^2^Department of Biological Sciences, Graduate School of Sciences and Technology for Innovation, Yamaguchi University, Yamaguchi, Japan; ^3^Department of Veterinary Pathophysiology and Animal Health, Graduate School of Agricultural and Life Sciences, The University of Tokyo, Bunkyo, Japan; ^4^Racehorse Hospital, Miho Training Center, Inashiki, Japan

**Keywords:** heat stress, exercise, Thoroughbred horses, messenger RNA, skeletal muscle

## Abstract

Heat acclimatization or acclimation training in horses is practiced to reduce physiological strain and improve exercise performance in the heat, which can involve metabolic improvement in skeletal muscle. However, there is limited information concerning the acute signaling responses of equine skeletal muscle after exercise in a hot environment. The purpose of this study was to investigate the hypothesis that exercise in hot conditions induces greater changes in heat shock proteins and mitochondrial-related signaling in equine skeletal muscle compared with exercise in cool conditions. Fifteen trained Thoroughbred horses [4.6 ± 0.4 (mean ± SE) years old; 503 ± 14 kg] were assigned to perform a treadmill exercise test in cool conditions [*COOL*; Wet Bulb Globe Temperature (WBGT), 12.5°C; *n* = 8] or hot conditions (*HOT*; WBGT, 29.5°C; *n* = 7) consisting of walking at 1.7 m/s for 1 min, trotting at 4 m/s for 5 min, and cantering at 7 m/s for 2 min and at 90% of *V*O_2max_ for 2 min, followed by walking at 1.7 m/s for 20 min. Heart rate during exercise and plasma lactate concentration immediately after exercise were measured. Biopsy samples were obtained from the middle gluteal muscle before and at 4 h after exercise, and relative quantitative analysis of mRNA expression using real-time RT-PCR was performed. Data were analyzed with using mixed models. There were no significant differences between the two groups in peak heart rate (*COOL*, 213 ± 3 bpm; *HOT*, 214 ± 4 bpm; *p* = 0.782) and plasma lactate concentration (*COOL*, 13.1 ± 1.4 mmoL/L; *HOT*, 17.5 ± 1.7 mmoL/L; *p* = 0.060), while HSP-70 (*COOL*, 1.9-fold, *p* = 0.207; *HOT*, 2.4-fold, *p* = 0.045), PGC-1α (*COOL*, 3.8-fold, *p* = 0.424; *HOT*, 8.4-fold, *p* = 0.010), HIF-1α (*COOL*, 1.6-fold, *p* = 0.315; *HOT*, 2.2-fold, *p* = 0.018) and PDK4 (*COOL*, 7.6-fold, *p* = 0.412; *HOT*, 14.1-fold, *p* = 0.047) mRNA increased significantly only in *HOT* at 4 h after exercise. These data indicate that acute exercise in a hot environment facilitates protective response to heat stress (HSP-70), mitochondrial biogenesis (PGC-1α and HIF-1α) and fatty acid oxidation (PDK4).

## 1. Introduction

Heat acclimatization or acclimation training has generally been practiced in humans to reduce physiological strain, decrease aerobic metabolic rate, and improve exercise performance in the heat (1–5). Studies in humans and rodents have shown that these adaptations involve changes at the cellular level, including improved cytoprotection against heat stress, stimulated mitochondrial biogenesis, enhanced fatty acid oxidation, and angiogenesis promotion (6–9).

Heat shock proteins (HSPs) are classified as molecular chaperones that play a central role in the cellular stress response to a hot environment, and increased expression of HSPs has a protective function against cell injury associated with adverse stresses (10, 11). In addition, Henstridege et al. demonstrated that genetic overexpression of HSP-70 upregulates oxidative metabolism in rodent skeletal muscle and that HSP-70 may play an important role in the regulation of mitochondrial biogenesis (12). HSP-90, a member of the HSPs, also has specific characteristics and stimulates the activation of signaling transduction pathways distinct from those stimulated by HSP-70. Katschinski et al. demonstrated that the chaperone activity of HSP-90 is involved in hypoxic-inducible factor-1α (HIF-1α) stabilization *in vitro* (13). HIF-1α regulates vascular endothelial growth factor (VEGF), which is implicated as important modulator of angiogenesis and facilitates migration. Both HSP-70 and HSP-90 augment proportionally increased cellular heat stress (14), in particular, HSP-70 gene expression increases synergistically when combined with exercise and hot conditions in rats (15). In addition, Morton et al. indicated that there is a sex specific HSP adaptation caused by estrogen in human skeletal muscle (16), and Gillum et al. have demonstrated that male increased HSP-72 more than female in peripheral blood mononuclear cells in response to running at 65% *V*O_2peak_ for 60 min in the heat (ambient temperature, 42°C; relative humidity, 20%) (17). In contrast, Mee et al. have demonstrated that there is no sex difference in leucocyte HSP72 mRNA after cycling at 65% *V*O_2peak_ for 90 min under a hot environment (ambient temperature, 40°C; relative humidity, 40%) in humans (18). However, there is limited information concerning the sex differences on HSPs in Thoroughbred horses.

Several studies have demonstrated that chronic heat stress can upregulate mitochondrial-related factors *in vitro* and *in vivo*, including humans and rodents. Repeated exposure to high temperature (40°C) can induce mitochondrial biogenesis and mitochondrial oxidative efficiency in C2C12 myotubes (19, 20). In addition, Maunder et al. demonstrated that endurance training under environmental heat stress (ambient temperature, 33°C; relative humidity, 80%) improves endurance performance and induces mitochondrial adaptation in humans (6). The increased energy demands associated with physiological strain activate the expression of the muscle peroxisome proliferator-activated receptor γ coactivator-1α (PGC-1α), a master regulator of mitochondrial biogenesis, gene expression, which can induce pyruvate dehydrogenase kinase 4 (PDK4) gene expression *via* estrogen-related receptor α (21). PDK4 facilitates the inactivation of pyruvate dehydrogenase complex, promoting an increase in mitochondrial fatty acid oxidation and a concurrent decrease in glucose oxidation (22). In terms of acute exercise, Tamura et al. demonstrated that post-exercise in the chamber where ambient temperature regulated 40°C upregulates PGC-1α expression in mouse skeletal muscle (7). In contrast, Heesch et al. demonstrated that acute exercise in a hot environment (ambient temperature, 33°C; relative humidity, 60%) blunts mitochondrial biogenesis related gene expression both immediately and at 3 h postexercise in humans (23). Therefore, the effect of acute exercise in a hot environment on mitochondrial adaptations is still controversial and needs further investigation.

Thoroughbreds have a high capacity for energy production and produce heat that elevates core temperature by approximately 1°C per minute during strenuous exercise (24). Furthermore, when these horses exercise in hot and humid environments (ambient temperature, 32–24°C; relative humidity, 80–85%), including racing or competition during daytime in summer, where heat dissipation is challenging, their core and muscle temperatures can rise to over 42°C during high-intensity exercise (25). Previously, we reported that exercise in an environment with an ambient temperature that exceeds body temperature causes a significant elevation of core body temperature and leads to decreased running economy (26). In addition, several other studies have demonstrated that exercise in a hot environment increases physiological strain and impairs exercise performance (25, 27). As a countermeasure to reduce these deleterious effects of heat stress, it is recommended that horses train in hot conditions similar to human athletes (28), and several studies have demonstrated that repeated exposure to heat stress can improve aerobic exercise performance and reduces metabolic rate in horses (29, 30). Therefore, we hypothesized that heat acclimatization or acclimation training would induce not only physiological adaptations but also cellular adaptations in skeletal muscle in horses. Several studies reported that the mRNA expression of heat shock proteins and mitochondria-associated proteins is increased after acute exercise in temperate conditions (31–33) and that there are no sex differences in the enzyme activities related to oxidative capacity in equine skeletal muscle (34). However, there is limited information concerning the differences in the acute signaling responses of equine skeletal muscle after exercise in a hot environment compared to exercise in a cool environment. Understanding these skeletal muscle responses can help construct a training strategy for races and competitions in a hot environment.

The purpose of this study was to investigate the hypothesis that exercise in hot conditions induces greater changes in heat shock proteins and mitochondrial-related signaling in equine skeletal muscle compared to exercise in cool conditions.

## 2. Materials and methods

### 2.1. Animals

Fifteen Thoroughbred horses [one male, seven geldings and seven females; age, 4.6 ± 0.4 (mean ± SE) years; body weight, 503 ± 14 kg; maximal oxygen consumption (*V*O_2max_), 174 ± 7 (ml/min kg)] were studied. Their characteristics are presented in Table 1. This study was conducted in Shimotsuke city (Japan) from February to March (average ambient temperature: 5–10°C) when the horses were not acclimatized to hot conditions. Horses had moderate-intensity training (1.7 m/s for 2 min, 4.0 m/s for 5 min, 7.0 m/s for 2 min, and 10.0 m/s for 2 min) on a treadmill (SÄTO AB, Knivsta, Sweden) at a 6% incline in cool conditions [ambient temperature, 20°C; relative humidity, 40%; Wet Bulb Globe Temperature (WBGT), 15°C; Temperature Humidity Index (THI), 62.8] 2 days/week for 4 weeks and walked for 1 h/day in a walker for 5 days/week and were kept in a 400 m^2^ paddock for 6 h/day prior to the experiment. After training, the horses performed preliminary incremental exercise tests in cool conditions to determine their *V*O_2max_. Following a warm up at 4 m/s for 3 min, the horses began exercising up a 6% incline for 2 min each at 1.7, 4, 6, 8, 10, 12 and 13 m/s until the houses could not maintain their position at the front of the treadmill with human encouragement.

**Table 1:** Characteristics of fifteen horses used in the present study.

	Age (years)	Weight (kg)	*V*O_2max_ (ml/min kg)
Male (*n* = 1)	4	513	164
Gelding (*n* = 7)	5.1 ± 0.4	528 ± 24	174 ± 8
Female (*n* = 7)	3.9 ± 0.6	477 ± 16	168 ± 5

### 2.2. Oxygen consumption

The procedure for measuring oxygen consumption has been described previously (35–37). Horses wore a 25 cm diameter open-flow mask on the treadmill, with a rheostat-controlled blower drawing air. Air flowed through 25 cm diameter tubing and across a pneumotachograph (LF-150B, Vise Medical, Chiba, Japan) connected to a differential pressure transducer (TF-5, Vise Medical, Chiba, Japan); this ensured that the bias flows during measurements were identical to those used during calibrations. Oxygen and CO_2_ concentrations were measured by an O_2_ and CO_2_ analyzer (MG-360, Vise Medical, Chiba, Japan), and calibrations were performed to calculate rates of O_2_ consumption and CO_2_ production with electronic mass flow meters (CR-300, Kofloc, Kyoto, Japan) using the N_2_-dilution/CO_2_-addition mass-balance technique (38). Gas analyzer and mass flow meter outputs were recorded on personal computer and analyzed with commercial hardware and software (DI-720 and Windaq Pro+, DATAQ, Akron, OH). *V*O_2_ was calculated for the final 30 s of each step at the incremental exercise test.

### 2.3. Experimental design

Horses were assigned to two groups, horses exercising in cool conditions (*COOL*, *n* = 8) or horses exercising in hot conditions (*HOT*, *n* = 7), to match their run time and *V*O_2max_ from the preliminary incremental exercise test. Each horse performed a treadmill exercise test consisting of walking at 1.7 m/s for 1 min, trotting at 4 m/s for 5 min, cantering at 7 m/s for 2 min, and eliciting 90% *V*O_2max_ (10.4 ± 0.2 m/s) for 2 min, followed by walking at 1.7 m/s for 20 min in either cool conditions (*COOL* group, ambient temperature, 18.1 ± 0.5°C; relative humidity, 28.0 ± 2.5%; WBGT, 12.5 ± 0.5°C; THI, 61.2 ± 0.4) or hot conditions (*HOT* group, ambient temperature, 38.2 ± 0.4°C; relative humidity, 31.7 ± 0.4%; WBGT, 29.5 ± 0.3°C; THI, 77.3 ± 0.3). The room temperature was controlled using air conditioners (RAS-AP140DG4, Hitachi, Tokyo, Japan) and oil heaters (HPS360, Orion, Nagano, Japan). Ambient temperature, relative humidity, and WBGT were measured using a portable monitoring device (WBGT-213B, Kyoto Electronics Manufacturing, Kyoto, Japan).

### 2.4. Heart rate and plasma lactate concentration

A heart rate monitor (S810, Polar, Kempele, Finland) was attached around the thorax and mean heart rate was calculated for the final 30 s of each step of the exercise test. Venous blood was collected from the jugular vein *via* an 18-gauge needle into a heparin tube immediately after exercise. Blood samples were centrifuged (AX-511, Tomy industrial, Tokyo, Japan) at 1740 ×*g* for 10 min to measure plasma lactate concentration with a lactate analyzer (Biosen S-Line, EKF-diagnostic GmbH, Barleben, Germany).

### 2.5. Muscle biopsy

Before exercise (pre) and 4 h after exercise, muscle samples (~50 mg wet weight) were obtained from the same area (the two sampling points were approximately 2 cm apart) at the mid-section of the *gluteus medius* muscle and from the same depth (5 cm below the skin surface) by needle biopsy under local anesthesia (Lidocaine, Fujisawa pharmaceutical, Osaka, Japan). All muscle samples were immediately frozen by liquid nitrogen and stored at −80°C until analyzed.

### 2.6. RNA isolation and real time RT-PCR

The procedure for RT-PCR has been described previously (39). Total RNA was extracted from each muscle sample with a TRIZOL regimen (Molecular Probes, Breda, Netherlands). The purity and quantity of total RNA were determined by measuring the absorbance of aliquots at 260 and 280 nm. Total RNA was then treated for 30 min at 37°C with TURBO DNase (Ambion, Austin, TX, United States) to remove genomic DNA from samples. DNase-treated RNA (0.5 μg) was used to synthesize first-stand cDNA with an Exscript™ RT reagent Kit (Takara, Tokyo, Japan). Thereafter, the cDNA products were analyzed by real-time PCR using the SYBR Green PCR Master Mix protocol in a StepOne™ Real Time PCR System (Applied Biosystems Japan, Tokyo, Japan).

The amplification program included an initial denaturation step at 95°C for 10 min, 40 cycles of denaturation at 95°C for 30 s, and annealing/extension at 58°C for 1 min. The amount of glyceraldehyde-3-phosphate dehydrogenase (GAPDH) mRNA was estimated as an internal control (40). Each mRNA was normalized to GAPDH by subtracting the cycle threshold (Ct) value of GAPDH from the Ct value of the gene target [ΔCt (target)]. The relative expression of the target gene was calculated as the relative quantification value for the pre value. Following the relative expression, dissociation curve analysis detected no nonspecific amplification in cDNA samples.

The sequences of the specific primers used in this study are presented in Table 2. Each PCR primer was designed by Primer Express® software (Applied Biosystems Japan, Tokyo, Japan), and oligonucleotides were purchased from FASMAC (FASMAC, Kanagawa, Japan).

**Table 2:** Real-time reverse transcription (RT)-PCR primer sequences.

	Forward sequence	Reverse sequence
GAPDH	CAAGGCTGTGGGCAAGGT	GGAAGGCCATGCCGTGA
HSP-70	CATCCTGAACGTCACGGCCAC	CCTTCTTCTTGTC(C/A/G)GCCTCG
HSP-90	CCCTGTTGTGGCGTGTGA	CGAGCACTACAGGGCAAGGT
HIF-1α	AAGTGCGAGCACGATTACAGTAT	GACGGTAGGAAGAGCAGGTTCTT
VEGF	CCCACTGCGGAGTTCAACAT	TTGGCTTTGGTGAGGTTTGAT
ANGPT1	GCAAATGTGCCCTCATGCT	CAGATTGGATGGGCCACAAG
PGC-1α	TCCGTGTCACCACCCAAAT	TGAACGAGAGCGCATCCTT
PDK4	GCTGGTTTTGGTTATGGCTTGC	TCCACAGACTCAGAAGACAAAGCC
SDHa	AGGTTTGCTGATGGCAGTATAAGA	TGCATCGACTTCTGCATGCT
PFK	GGTGGCACAGTGATTGGAAGT	CGGAGTCGTCCCTCTCGTT
MCT1	GATTCTTGGCGGCTGCTTGTCAGG	TGCCAATCATGGTCAGAGCCGGA
MCT4	ATGGTGTCTGCGTCCTTCTGCGGA	AGCGCCAAACCCAAGCCGGTAA
CD31	GCAGATGATTCCTGTGTTCCAA	TGGGAGCAGGGCAGGTT

GAPDH, glyceraldehydes-3-phosphate dehydrogenase; HSP-70, heat shock protein 70; HSP-90, heat shock protein 90; HIF-1α, hypoxia inducible factor-1α; VEGF, vascular endothelial growth factor; ANGPT1, angiopoietin-1; PGC-1α, peroxisome proliferator-activated receptor γ coactivator-1α; PDK4, pyruvate dehydrogenase kinase 4; SDHa, succinate dehydrogenase complex flavoprotein subunit A; PFK, phosphofructokinase; MCT1, monocarboxylate transporter 1; MCT4, monocarboxylate transporter 4; CD31, cluster of differentiation 31.

### 2.7. Statistical procedures

All data are presented as mean ± standard error (SE). Differences in heart rate and plasma lactate concentration between *COOL* and *HOT* during exercise were analyzed using Student’s *t*-test. We analyzed mRNA expressions using mixed model with time, group and sex as fixed effects and individual horse as a random effect. When a significant effect or interaction was observed, Tukey’s tests were used as *post hoc* tests. For all analyses, significance was set as *p* ≤ 0.05. Statistical software (JMP 16.2.0, SAS Institute Inc., Cary, NC) was used for all data analyses.

## 3. Results

### 3.1. Heart rate and plasma lactate concentration

**Figure 1:**
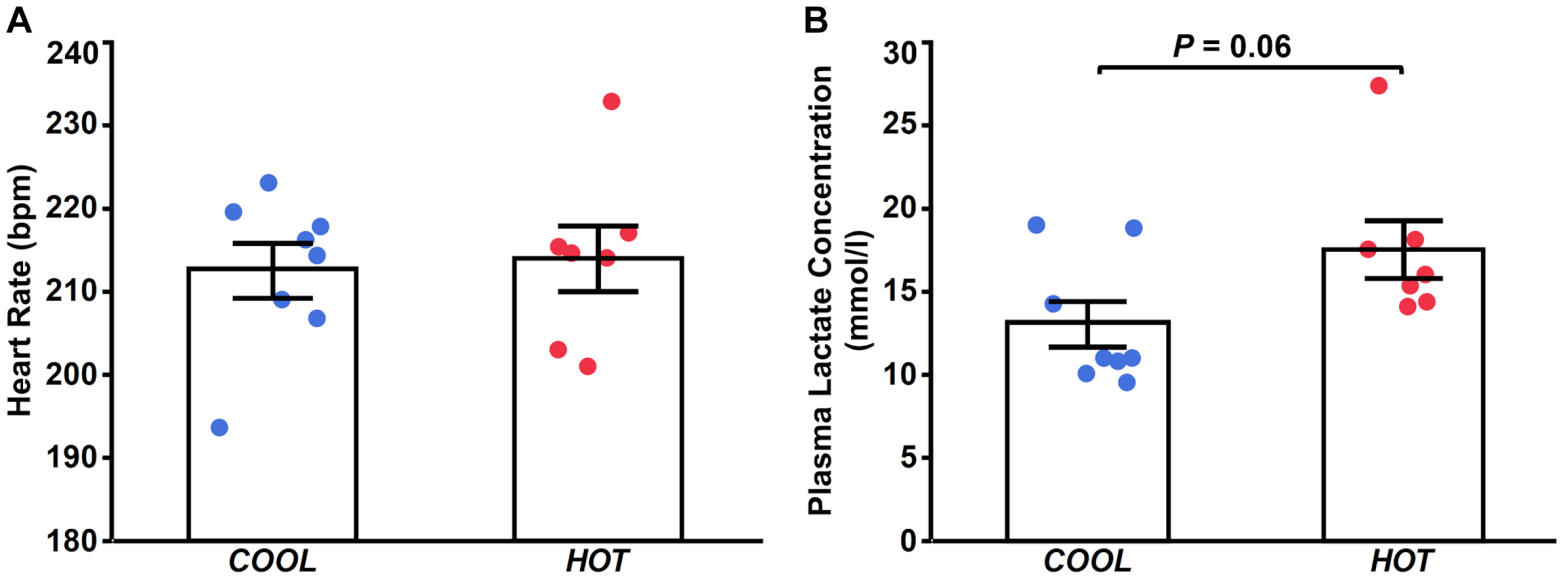
Heart rate **(A)** at the end of exercise and plasma lactate concentration immediately after exercise **(B)** in *COOL* (blue) and *HOT* (red).

There were no differences between groups in peak heart rate during exercise (*COOL*, 213 ± 3 bpm; *HOT*, 214 ± 4 bpm; *p* = 0.782; Figure 1A). Plasma lactate concentration immediately after exercise in *HOT* tended to be higher than that in *COOL*, but the difference was not significant (*COOL*, 13.1 ± 1.4 mmoL/L; *HOT*, 17.5 ± 1.7 mmoL/L; *p* = 0.060; Figure 1B).

### 3.2. Gene expression

**Figure 2:**
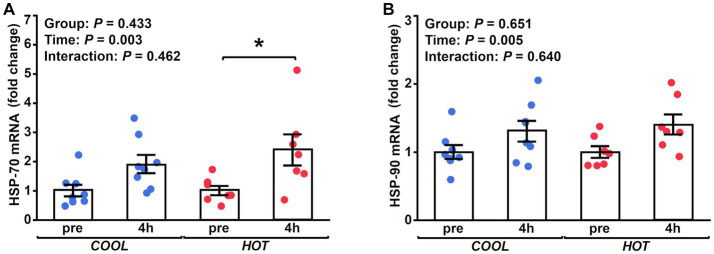
Fold changes in the mRNA expression of HSP-70 **(A)** and HSP-90 **(B)** at 4 h after exercise compared to before exercise (pre) in *COOL* (blue) and *HOT* (red). *Significant differences from pre (*p* < 0.05).

**Figure 3:**
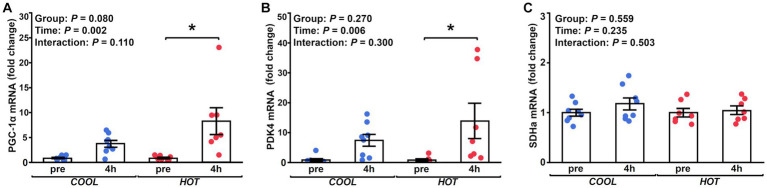
Fold changes in the mRNA expression of PGC-1α **(A)**, PDK4 **(B)**, and SDHa **(C)** at 4 h after exercise compared to before exercise (pre) in *COOL* (blue) and *HOT* (red). *Significant differences from pre (*p* < 0.05).

**Figure 4:**
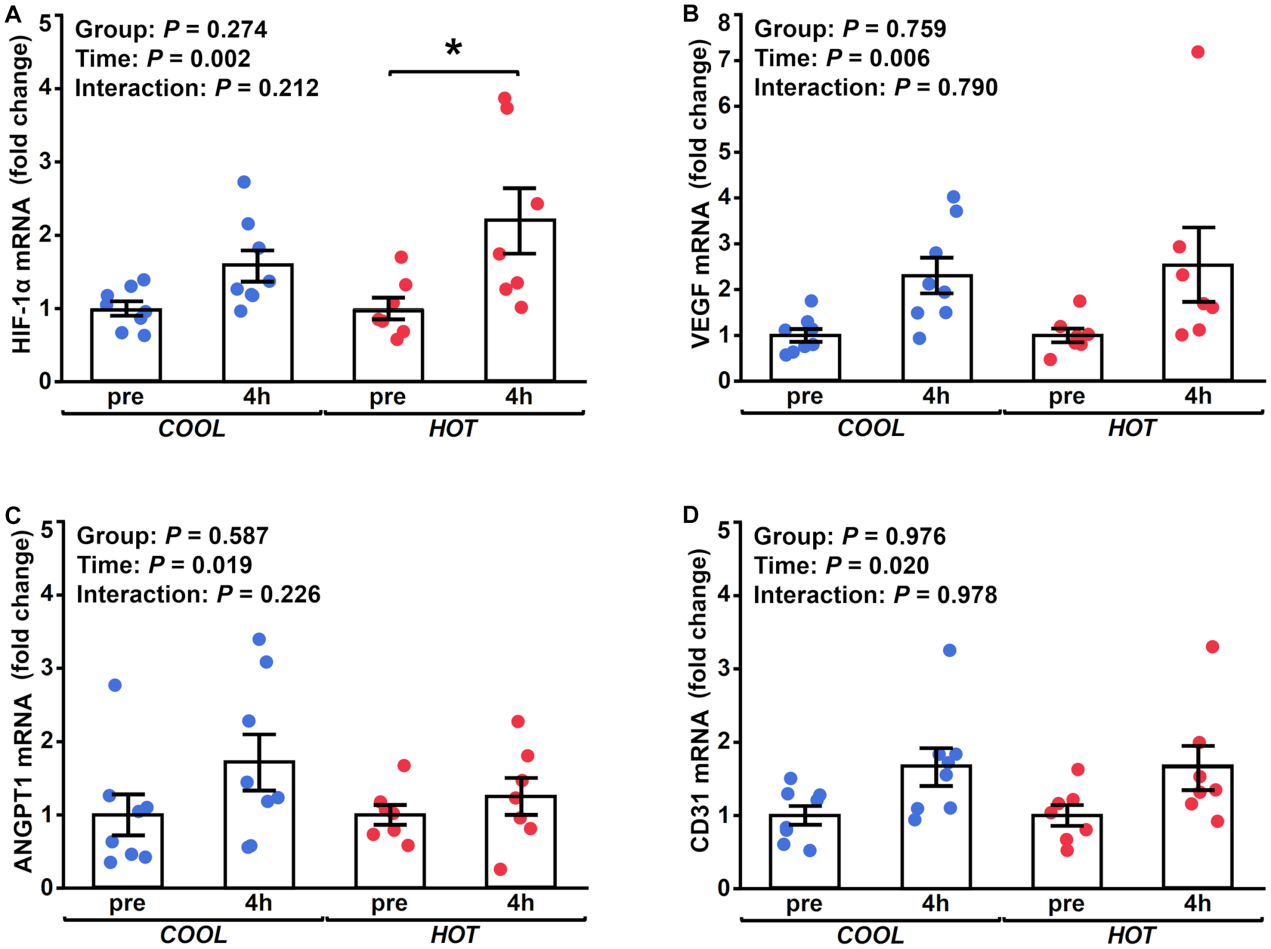
Changes in the mRNA expression of HIF-1α **(A)**, VEGF **(B)** and ANGPT1 **(C)**, and CD31 **(D)** at 4 h after exercise compared to before exercise (pre) in *COOL* (blue) and *HOT* (red). *Significant differences from pre (*p* < 0.05).

**Figure 5:**
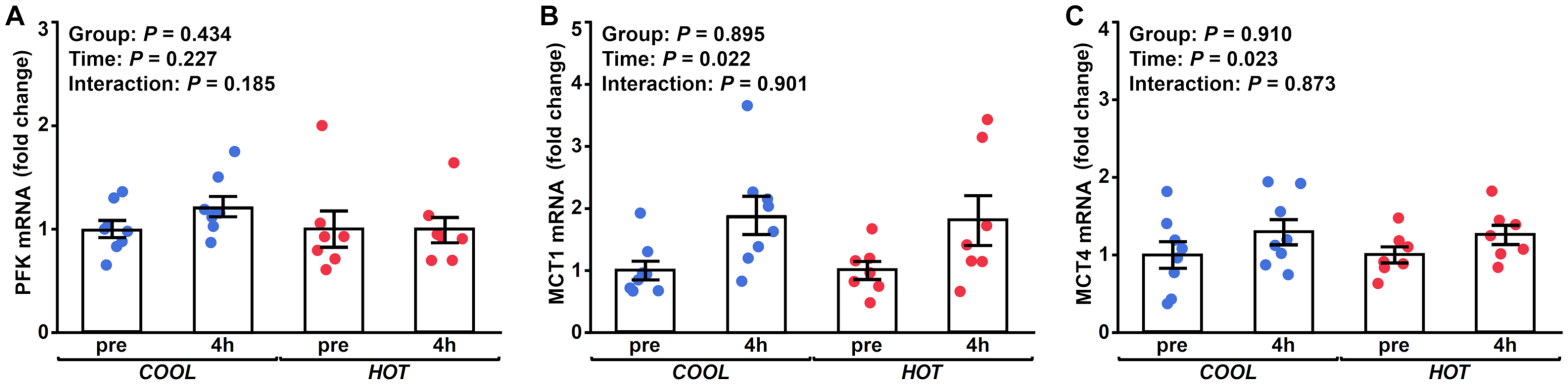
Changes in the mRNA expression of PFK **(A)**, MCT1 **(B)**, and MCT4 **(C)** at 4 h after exercise compared to before exercise (pre) in *COOL* (blue) and *HOT* (red).

HSP-70 mRNA expression demonstrated a significant time effect (2.1-fold, *p* = 0.003), but not a group effect (*p* = 0.433) or interaction (*p* = 0.462, Figure 2A). At 4 h after exercise, HSP-70 mRNA increased significantly only in *HOT* compared to pre (2.4-fold, *p* = 0.045), but not in *COOL* (1.9-fold, *p* = 0.207). In contrast, HSP-90 mRNA did not change in either group at 4 h (*COOL*, 1.3-fold, *p* = 0.189; *HOT*, 1.4-fold, *p* = 0.080, Figure 2B), although a time effect was observed (1.4-fold, *p* = 0.005) similar to HSP-70. PGC-1α mRNA demonstrated a significant time effect (6.0-fold, *p* = 0.002), but not a group effect (*p* = 0.080) or interaction (*p* = 0.110, Figure 3A). PGC-1α mRNA levels increased in *HOT* (8.4-fold, *p* = 0.010) at 4 h compared to pre, but not in *COOL* (3.8-fold, *p* = 0.424). PDK4 mRNA expression demonstrated a significant time effect (10.6-fold, *p* = 0.006), but not a group effect (*p* = 0.270) or interaction (*p* = 0.300, Figure 3B). In addition, an increase in PDK4 mRNA was observed only in *HOT* at 4 h compared to pre (14.1-fold, *p* = 0.047), but not in *COOL* (7.6-fold, *p* = 0.412). SDHa mRNA expression did not reveal a significant time effect (1.1-fold, *p* = 0.235), group effect (*p* = 0.559), or interaction (*p* = 0.503, Figure 3C). HIF-1α mRNA expression demonstrated a significant time effect (1.9-fold, *p* = 0.002), but not a group effect (*p* = 0.274) or interaction (*p* = 0.212, Figure 4A). A significant increase in HIF-1α mRNA levels was observed only in *HOT* at 4 h compared to pre (2.2-fold, *p* = 0.018), but not in *COOL* (1.6-fold, *p* = 0.315). VEGF, ANGPT1 and CD31 mRNA expressions demonstrated significant time effects (2.4-fold, *p* = 0.006, 1.5-fold, *p* = 0.019, 1.7-fold, *p* = 0.020, respectively; Figures 4B–D), but did not change at 4 h compared to pre in either group (VEGF: *COOL*, 2.3-fold, *p* = 0.293; *HOT*, 2.5-fold, *p* = 0.136; ANGPT1: *COOL*, 1.7-fold, *p* = 0.221; *HOT*, 1.3-fold, *p* = 0.901; CD31: *COOL*, 1.7-fold, *p* = 0.122; *HOT*, 1.7-fold, *p* = 0.153). PFK mRNA did not show a significant time effect (1.1-fold, *p* = 0.227), group effect (*p* = 0.434), or interaction (*p* = 0.185, Figure 5A). MCT1 and MCT4 mRNA expression demonstrated a significant time effect (1.8-fold, *p* = 0.022; 1.3-fold, *p* = 0.023, respectively; Figures 5B,C) but did not change in both groups at 4 h compared to pre (MCT1: *COOL*, 1.9-fold, *p* = 0.067; *HOT*, 1.6-fold, *p* = 0.146; MCT4: *COOL*, 1.3-fold, *p* = 0.442; *HOT*, 1.3-fold, *p* = 0.767). We did not observe any significant effects on sex in any valuables (HSP-70, *p* = 0.268; HSP-90, *p* = 0.184; PGC-1α, *p* = 0.164; PDK4, *p* = 0.446; SDHa, *p* = 0.803; HIF-1α, *p* = 0.461; VEGF, *p* = 0.424; ANGPT1, *p* = 0.245; CD31, *p* = 0.414; PFK, *p* = 0.358; MCT1, *p* = 0.374; MCT4, *p* = 0.720).

## 4. Discussion

Our main finding in this study is that exercise in a hot environment upregulates the expression of HSP-70 mRNA and PGC-1α mRNA in Thoroughbred horses. These results indicate that the combination of heat exposure and exercise facilitate the activation of heat stress responses and mitochondrial adaptations in Thoroughbred horses.

HSP-70 is classified as molecular chaperone that plays a central role in the cellular stress response to hot environments and has a protective function against cell injury associated with adverse stresses (41). Previous studies have shown that acute thermal stress with an increase in body temperature enhances HSP-70 mRNA expression in various species, including humans and rodents (7, 15, 42, 43). Heat shock factor 1, which is a direct transcriptional activator that induces heat shock gene transcription following heat stress, directly activates the expression of PGC-1α, a master regulator in mitochondrial biogenesis (44, 45). Although we did not measure body core temperature during exercise in this study, previous studies have demonstrated that pulmonary artery temperature during exercise under hyperthermic conditions (WBGT 28°C) is 1–2°C higher than that under cool conditions (WBGT 15°C) in horses (25–27). Therefore, it is reasonable to assume that core and muscle temperature in *HOT* was also higher than that in *COOL*, inducing sufficient thermal stress to upregulate the expression of these genes. Furthermore, the expression of PGC-1α mRNA is enhanced in an intensity-dependent manner (46–48). In horses, several studies have demonstrated that *V*O_2_ during submaximal exercise in hot conditions is higher compared to that at the same speed in cool conditions (26, 27), and increased relative workload may also contribute the upregulation of PGC-1α mRNA. Therefore, additional increases in core and muscle temperatures with the combination of heat exposure and exercise enhance HSP-70 and PGC-1α mRNA responses, which may be beneficial for cytoprotection and mitochondrial biogenesis.

While the expression of HSP-70 mRNA increased following exercise, no significant change was observed in HSP-90 mRNA. HSP-90, similar to HSP-70, plays a crucial role in cytoprotection and acts as biochemical sensors for various stressors, including heat shock and exercise (41). The extent of HSPs induction following a single bout of exercise depends on several factors, including exercise modality, duration, intensity, and training status of the subject (41, 49). Skidmore et al. demonstrated that a combination of exercise and a hot environment increases synergistic HSP-70 induction compared to either alone in rats (15). In addition, Cuthbert et al. demonstrated that HSP-70 mRNA increases 2- to 4-fold after moderate-intensity exercise (60% *V*O_2peak_) in trained humans, while HSP-90 mRNA remains unchanged. Our findings are consistent with the results of Cuthbert et al. that indicate that the sensitivity response to stress differs depending of the type of HSP and that HSP-70 may be a more sensitive marker of heat stress compared with HSP-90 in trained Thoroughbred horses.

In the present study, we detected increased PDK4 mRNA coincident with increased PGC-1α mRNA in Thoroughbred horses training in hot conditions (21, 22). Several studies have demonstrated that muscle glycogen utilization is reduced with heat acclimation (8, 50). Thus, the improved muscle energy metabolism with heat acclimation may involve a shift in energy fuel utilization from glucose oxidation to fatty acid oxidation due to the upregulation of PDK4.

In a hot environment, cutaneous blood flow increases through peripheral vasodilation for heat dissipation in horses and humans (51, 52). During prolonged low-to-moderate intensity exercise, muscle blood flow is not reduced because an increase in cardiac output can compensate the increased skin blood flow (2, 53). However, González-Alonso and Calbet demonstrated that blood flow to exercising muscle is reduced during high-intensity exercise (cycling at 80% of peak power output) starting with high skin and core temperature in humans (54). Therefore, the blood flow demands from skin and muscle may exceed the cardiac capacity, and blood flow to skeletal muscle may have reduced during high-intensity exercise (90% *V*O_2_max) in this study. HIF-1α activates in response to hypoxia in various tissues, including skeletal muscle, and plays an integral role in regulating the various genes involved in the hypoxic response (55). Reduced blood flow to skeletal muscle during intense exercise in hot conditions may cause more severe hypoxia in skeletal muscle tissue. Therefore, exercise in a hot environment may result in cellular adaptation that counteracts the effects of reduced oxygen supply to cells by activating HIF-1α. While we observed increased HIF-1α mRNA, no changes were detected in VEGF, ANGPT1, and CD31 mRNA levels in each group. VEGF, ANGPT1, and CD31 are factors related to angiogenesis and are located downstream of HIF-1α. In contrary to our findings in this study, Gustafsson et al. demonstrated that a single bout of cycling increased VEGF mRNA in human skeletal muscle and that this increase positively correlated with the change in HIF-1α mRNA (56). Additionally, Gustafsson et al., also demonstrated a correlation between the increase in VEGF mRNA and plasma lactate concentration after exercise (56). In humans, exercise in a hot environment often enhances glycolysis in skeletal muscle, which may be probably due to reduced blood flow and oxygen delivery to working muscle (57). In this study, we observed no differences in the plasma lactate concentration between *HOT* and *COOL*. Therefore, the reduction in muscle blood flow and HIF-1α induction may be insufficient to enhance angiogenesis in this study.

We observed no changes in PFK, MCT1, and MCT4 mRNA expressions in this study. PFK is a kinase enzyme that phosphorylates fructose 6-phosphate and is the main rate-controlling enzyme of glycolysis. MCT1 and 4 are key factors in mediating lactate transport. There were no differences in the plasma lactate concentration between *HOT* and *COOL*, which is reasonable in that gene expressions related to the glycolysis pathway and lactate transport were not enhanced in *HOT*. Previous studies in humans and horses have showed an augmented intramuscular carbohydrate utilization and an increased blood lactate concentration when comparing prolonged submaximal exercise in a hot environment with that in a cool environment (1, 27, 58). In contrast, Maxwell et al. demonstrated no differences in the rate of muscle glycogenolysis and blood lactate accumulation during intermittent high-intensity exercise between hot and cool environments (59, 60). These results, including our results, indicate that the increased glycolysis and blood lactate accumulation may depend on several factors, including the duration and intensity of exercise, training status of subjects, environmental temperature, and the timing of sample collection.

We acknowledge there are limitations in this study. First, we obtained muscle samples only at 4 h after exercise and did not observe time-course changes of gene expressions over time. Because the time-course changes of gene expressions vary with target markers (61), we could detect more mRNA changes if we collected muscle samples at several time points. Second, we only observed changes in mRNA expression following acute exercise. In humans, 10 days or longer heat acclimation are recommended to induce optimize adaptations (62). Some reports in horses have demonstrated that heat acclimation have beneficial effects on physiological responses similar to humans (29, 63). However, these studies did not evaluate cellular adaptations in skeletal muscle. Therefore, further studies are necessary to fully investigate the long-term effects of heat acclimatization or acclimation on skeletal muscle adaptations.

In summary, we have demonstrated that HSP-70, PGC-1α, HIF-1α, and PDK4 mRNA significantly increased after high-intensity exercise in hot conditions. These increases in gene expressions may facilitate a protective response to heat stress, mitochondrial biogenesis, and fatty acid oxidation. Therefore, our results suggest that heat acclimatization or acclimation for competitions and races in hot conditions is effective in improving aerobic energy metabolism, as well as reducing heat stress. Repeated exposure to heat stress, such as training in natural hot environment or in artificial heated environment, may be effective strategy for competitions and races held in hot environments.

## Data availability statement

The original contributions presented in the study are included in the article/supplementary material, further inquiries can be directed to the corresponding author.

## Ethics statement

The animal study was reviewed and approved by the Animal Welfare and Ethics Committee of the Japan Racing Association’s Equine Research Institute (accession number 21-1).

## Author contributions

YE, KM, YT, and HO contributed to the conception and design of the study. YE, KM, YT, TY, AK, TM, HM, and HO carried out the experiments. YE, KM, YT, TY, and HO performed data collection. YE, AK, TM, and HM analyzed the data. YE wrote the first draft of the manuscript. All authors contributed to the article and approved the submitted version.

## Funding

The research was funded by the Japan Racing Association.

## Conflict of interest

YE, KM, YT, TY, and HO are employees of the Japan Racing Association.

The remaining authors declare that the research was conducted in the absence of any commercial or financial relationships that could be construed as a potential conflict of interest.

## Publisher’s note

All claims expressed in this article are solely those of the authors and do not necessarily represent those of their affiliated organizations, or those of the publisher, the editors and the reviewers. Any product that may be evaluated in this article, or claim that may be made by its manufacturer, is not guaranteed or endorsed by the publisher.

## References

[1] Febbraio MA Snow RJ Hargreaves M Stathis CG Martin IK Carey MF. Muscle metabolism during exercise and heat stress in trained men: effect of acclimation. J Appl Physiol (1985). (1994) 76:589–97. doi: 10.1152/jappl.1994.76.2.589, PMID: 8175568

[2] Nielsen B Hales JR Strange S Christensen NJ Warberg J Saltin B. Human circulatory and thermoregulatory adaptations with heat acclimation and exercise in a hot, dry environment. J Physiol. (1993) 460:467–85. doi: 10.1113/jphysiol.1993.sp019482, PMID: 8487204 PMC1175224

[3] Lorenzo S Halliwill JR Sawka MN Minson CT. Heat acclimation improves exercise performance. J Appl Physiol (1985). (2010) 109:1140–7. doi: 10.1152/japplphysiol.00495.2010, PMID: 20724560 PMC2963322

[4] Keiser S Fluck D Huppin F Stravs A Hilty MP Lundby C. Heat training increases exercise capacity in hot but not in temperate conditions: a mechanistic counter-balanced cross-over study. Am J Physiol Heart Circ Physiol. (2015) 309:H750–61. doi: 10.1152/ajpheart.00138.2015 26150574

[5] Young AJ Sawka MN Levine L Cadarette BS Pandolf KB. Skeletal muscle metabolism during exercise is influenced by heat acclimation. J Appl Physiol (1985). (1985) 59:1929–35. doi: 10.1152/jappl.1985.59.6.1929, PMID: 4077800

[6] Maunder E Plews DJ Wallis GA Brick MJ Leigh WB Chang WL. Temperate performance and metabolic adaptations following endurance training performed under environmental heat stress. Physiol Rep. (2021) 9:e14849. doi: 10.14814/phy2.14849, PMID: 33977674 PMC8114151

[7] Tamura Y Matsunaga Y Masuda H Takahashi Y Takahashi Y Terada S. Postexercise whole body heat stress additively enhances endurance training-induced mitochondrial adaptations in mouse skeletal muscle. Am J Physiol Regul Integr Comp Physiol. (2014) 307:R931–43. doi: 10.1152/ajpregu.00525.2013 25080501

[8] Kirwan JP Costill DL Kuipers H Burrell MJ Fink WJ Kovaleski JE. Substrate utilization in leg muscle of men after heat acclimation. J Appl Physiol (1985). (1987) 63:31–5. doi: 10.1152/jappl.1987.63.1.31, PMID: 3624132

[9] Kuhlenhoelter AM Kim K Neff D Nie Y Blaize AN Wong BJ. Heat therapy promotes the expression of Angiogenic regulators in human skeletal muscle. Am J Physiol Regul Integr Comp Physiol. (2016) 311:R377–91. doi: 10.1152/ajpregu.00134.2016, PMID: 27357800 PMC5008657

[10] Kregel KC. Heat shock proteins: modifying factors in physiological stress responses and acquired Thermotolerance. J Appl Physiol (1985). (2002) 92:2177–86. doi: 10.1152/japplphysiol.01267.2001 11960972

[11] Moseley PL. Heat shock proteins and heat adaptation of the whole organism. J Appl Physiol (1985). (1997) 83:1413–7. doi: 10.1152/jappl.1997.83.5.1413 9375300

[12] Henstridge DC Bruce CR Drew BG Tory K Kolonics A Estevez E. Activating Hsp72 in rodent skeletal muscle increases mitochondrial number and oxidative capacity and decreases insulin resistance. Diabetes. (2014) 63:1881–94. doi: 10.2337/db13-0967, PMID: 24430435 PMC4030108

[13] Katschinski DM le L Heinrich D Wagner KF Hofer T Schindler SG. Heat induction of the Unphosphorylated form of hypoxia-inducible factor-1alpha is dependent on heat shock Protein-90 activity. J Biol Chem. (2002) 277:9262–7. doi: 10.1074/jbc.M110377200, PMID: 11779866

[14] McClung JP Hasday JD He JR Montain SJ Cheuvront SN Sawka MN. Exercise-heat acclimation in humans alters baseline levels and ex vivo heat Inducibility of Hsp72 and Hsp90 in peripheral blood mononuclear cells. Am J Physiol Regul Integr Comp Physiol. (2008) 294:R185–91. doi: 10.1152/ajpregu.00532.2007, PMID: 17977914

[15] Skidmore R Gutierrez JA Guerriero V Jr Kregel KC. Hsp70 induction during exercise and heat stress in rats: role of internal temperature. Am J Phys. (1995) 268:R92–7. doi: 10.1152/ajpregu.1995.268.1.R92 7840344

[16] Morton JP Holloway K Woods P Cable NT Burniston J Evans L. Exercise training-induced gender-specific heat shock protein adaptations in human skeletal muscle. Muscle Nerve. (2009) 39:230–3. doi: 10.1002/mus.21182, PMID: 19058194

[17] Gillum T Kuennen M Gourley C Dokladny K Schneider S Moseley P. Sex differences in heat shock protein 72 expression in peripheral blood mononuclear cells to acute exercise in the heat. Int J Endocrinol Metab. (2013) 11:e8739. doi: 10.5812/ijem.8739, PMID: 24719632 PMC3968984

[18] Mee JA Gibson OR Tuttle JA Taylor L Watt PW Doust J. Leukocyte Hsp72 Mrna transcription does not differ between males and females during heat acclimation. Temperature (Austin). (2016) 3:549–56. doi: 10.1080/23328940.2016.1214336, PMID: 28090558 PMC5198809

[19] Liu CT Brooks GA. Mild heat stress induces mitochondrial biogenesis in C2c12 Myotubes. J Appl Physiol (1985). (2012) 112:354–61. doi: 10.1152/japplphysiol.00989.2011, PMID: 22052865 PMC3774254

[20] Salgado RM Sheard AC Vaughan RA Parker DL Schneider SM Kenefick RW. Mitochondrial efficiency and exercise economy following heat stress: a potential role of uncoupling protein 3. Physiol Rep. (2017) 5:e13054. doi: 10.14814/phy2.13054, PMID: 28174343 PMC5309567

[21] Wende AR Huss JM Schaeffer PJ Giguere V Kelly DP. Pgc-1alpha Coactivates Pdk4 gene expression via the orphan nuclear receptor Erralpha: a mechanism for transcriptional control of muscle glucose metabolism. Mol Cell Biol. (2005) 25:10684–94. doi: 10.1128/MCB.25.24.10684-10694.2005, PMID: 16314495 PMC1316952

[22] Pettersen IKN Tusubira D Ashrafi H Dyrstad SE Hansen L Liu XZ. Upregulated Pdk4 expression is a sensitive marker of increased fatty acid oxidation. Mitochondrion. (2019) 49:97–110. doi: 10.1016/j.mito.2019.07.009, PMID: 31351920

[23] Heesch MW Shute RJ Kreiling JL Slivka DR. Transcriptional control, but not subcellular location, of Pgc-1alpha is altered following exercise in a hot environment. J Appl Physiol (1985). (2016) 121:741–9. doi: 10.1152/japplphysiol.01065.2015, PMID: 27445305 PMC5142252

[24] Hodgson DR Davis RE McConaghy FF. Thermoregulation in the horse in response to exercise. Br Vet J. (1994) 150:219–35. doi: 10.1016/S0007-1935(05)80003-X 8044664

[25] Geor RJ McCutcheon LJ Ecker GL Lindinger MI. Thermal and cardiorespiratory responses of horses to submaximal exercise under hot and humid conditions. Equine Vet J Suppl. (1995) 20:125–32. doi: 10.4085/1062-6050-50.9.0710.1111/j.2042-3306.1995.tb05018.x 8933095

[26] Ebisuda Y Mukai K Takahashi Y Ohmura H. Effect of high ambient temperature on physiological responses during incremental exercise in thoroughbred horses. Comparative Exercise Physiology. (2023) 19:159–67. doi: 10.3920/CEP220018

[27] MARLIN DJ SCOTT CM SCHROTER RC MILLS PC HARRIS RC HARRIS PA. Physiological responses in nonheat acclimated horses performing treadmill exercise in cool (20°C/40%RH), hot dry (30°C/40%RH) and hot humid (30°C/80%RH) conditions. Equine Vet J Suppl. (1996) 28:70–84. doi: 10.1111/j.2042-3306.1996.tb05034.x 8894553

[28] Hodgson DR. Thermoregulation In: Hodgson DR McKeever KH McGowan CM, editors. The Athletic Horse; Principles and Practice of Equine Sports Medicine. 2nd ed. St. Louis: Saunders-Elsevier (2014). 108–24.

[29] MARLIN DJ SCOTT CM SCHROTER RC HARRIS RC HARRIS PA ROBERTS CA. Physiological responses of horses to a treadmill simulated speed and endurance test in high heat and humidity before and after humid heat acclimation. Equine Vet J. (1999) 31:31–42. doi: 10.1111/j.2042-3306.1999.tb03788.x, PMID: 9952327

[30] Geor RJ McCutcheon LJ Ecker GL Lindinger MI. Heat storage in horses during submaximal exercise before and after humid heat acclimation. J Appl Physiol (1985). (2000) 89:2283–93. doi: 10.1152/jappl.2000.89.6.2283, PMID: 11090580

[31] Poso AR Eklund-Uusitalo S Hyyppa S Pirila E. Induction of heat shock protein 72 Mrna in skeletal muscle by exercise and training. Equine Vet J Suppl. (2002) 34:214–8. doi: 10.1111/j.2042-3306.2002.tb05421.x 12405689

[32] HILL EW EIVERS SS McGIVNEY BA FONSECA RG GU J SMITH NA. Moderate and high intensity Sprint exercise induce differential responses in Cox4i2 and Pdk4 gene expression in thoroughbred horse skeletal muscle. Equine Vet J Suppl. (2010) 42:576–81. doi: 10.1111/j.2042-3306.2010.00206.x, PMID: 21059063

[33] Eivers SS McGivney BA Fonseca RG MacHugh DE Menson K Park SD. Alterations in oxidative gene expression in equine skeletal muscle following exercise and training. Physiol Genomics. (2010) 40:83–93. doi: 10.1152/physiolgenomics.00041.2009, PMID: 19861432

[34] Roneus M Lindholm A Asheim A. Muscle characteristics in thoroughbreds of different ages and sexes. Equine Vet J. (1991) 23:207–10. doi: 10.1111/j.2042-3306.1991.tb02757.x, PMID: 1884703

[35] Jones JH Longworth KE Lindholm A Conley KE Karas RH Kayar SR. Oxygen transport during exercise in large mammals. I. Adaptive variation in oxygen demand. J Appl Physiol. (1989) 67:862–70. doi: 10.1152/jappl.1989.67.2.862, PMID: 2793686

[36] Birks EK Ohmura H Jones JH. Measuring Vo2 in hypoxic and Hyperoxic conditions using dynamic gas mixing with a flow-through indirect calorimeter. J Equine Sci. (2019) 30:87–92. doi: 10.1294/jes.30.87, PMID: 31871410 PMC6920056

[37] Mukai K Kitaoka Y Takahashi Y Takahashi T Takahashi K Ohmura H. Moderate-intensity training in hypoxia improves exercise performance and glycolytic capacity of skeletal muscle in horses. Physiol Rep. (2021) 9:e15145. doi: 10.14814/phy2.15145, PMID: 34889527 PMC8661515

[38] Fedak MA Rome L Seeherman HJ. One-step N2-dilution technique for calibrating open-circuit Vo2 measuring systems. J Appl Physiol. (1981) 51:772–6. doi: 10.4085/1062-6050-50.9.0710.1152/jappl.1981.51.3.772, PMID: 7327980

[39] Okabe K Mukai K Ohmura H Takahashi T Miyata H. Effect of acute high-intensity exercise in Normobaric hypoxia on thoroughbred skeletal muscle. J Sports Med Phys Fitness. (2017) 57:711–9. doi: 10.23736/S0022-4707.16.06154-5, PMID: 26955904

[40] Mahoney DJ Carey K Fu MH Snow R Cameron-Smith D Parise G. Real-time RT-PCR analysis of housekeeping genes in human skeletal muscle following acute exercise. Physiol Genomics. (2004) 18:226–31. doi: 10.1152/physiolgenomics.00067.2004, PMID: 15161965

[41] Kruger K Reichel T Zeilinger C. Role of heat shock proteins 70/90 in exercise physiology and exercise immunology and their diagnostic potential in sports. J Appl Physiol (1985). (2019) 126:916–27. doi: 10.1152/japplphysiol.01052.2018, PMID: 30730806

[42] Ihsan M Deldicque L Molphy J Britto F Cherif A Racinais S. Skeletal muscle signaling following whole-body and localized heat exposure in humans. Front Physiol. (2020) 11:839. doi: 10.3389/fphys.2020.00839, PMID: 32765299 PMC7381176

[43] Cuthbert RL Shute RJ Slivka DR. Skeletal muscle cold shock and heat shock protein Mrna response to aerobic exercise in different environmental temperatures. Temperature (Austin). (2019) 6:77–84. doi: 10.1080/23328940.2018.1555414, PMID: 30906813 PMC6422508

[44] Ma X Xu L Alberobello AT Gavrilova O Bagattin A Skarulis M. Celastrol protects against obesity and metabolic dysfunction through activation of a Hsf1-Pgc1alpha transcriptional Axis. Cell Metab. (2015) 22:695–708. doi: 10.1016/j.cmet.2015.08.005, PMID: 26344102

[45] Xu L Ma X Bagattin A Mueller E. The transcriptional coactivator Pgc1alpha protects against Hyperthermic stress via cooperation with the heat shock factor Hsf1. Cell Death Dis. (2016) 7:e2102. doi: 10.1038/cddis.2016.22, PMID: 26890141 PMC5399192

[46] Nordsborg NB Lundby C Leick L Pilegaard H. Relative workload determines exercise-induced increases in Pgc-1alpha Mrna. Med Sci Sports Exerc. (2010) 42:1477–84. doi: 10.1249/MSS.0b013e3181d2d21c, PMID: 20139785

[47] Brandt N Dethlefsen MM Bangsbo J Pilegaard H. Pgc-1alpha and exercise intensity dependent adaptations in mouse skeletal muscle. PLoS One. (2017) 12:e0185993. doi: 10.1371/journal.pone.0185993, PMID: 29049322 PMC5648136

[48] Egan B Carson BP Garcia-Roves PM Chibalin AV Sarsfield FM Barron N. Exercise intensity-dependent regulation of peroxisome proliferator-activated receptor Coactivator-1 Mrna abundance is associated with differential activation of upstream Signalling kinases in human skeletal muscle. J Physiol. (2010) 588:1779–90. doi: 10.1113/jphysiol.2010.188011, PMID: 20308248 PMC2887994

[49] Vogt M Puntschart A Geiser J Zuleger C Billeter R Hoppeler H. Molecular adaptations in human skeletal muscle to endurance training under simulated hypoxic conditions. J Appl Physiol (1985). (2001) 91:173–82. doi: 10.1152/jappl.2001.91.1.173, PMID: 11408428

[50] King DS Costill DL Fink WJ Hargreaves M Fielding RA. Muscle metabolism during exercise in the heat in Unacclimatized and acclimatized humans. J Appl Physiol (1985). (1985) 59:1350–4. doi: 10.1152/jappl.1985.59.5.1350, PMID: 4066564

[51] McConaghy FF Hodgson DR Rose RJ Hales JR. Redistribution of cardiac output in response to heat exposure in the pony. Equine Vet J Suppl. (1996) 22:42–6. doi: 10.1111/j.2042-3306.1996.tb05030.x, PMID: 8894549

[52] Johnson JM. Exercise in a hot environment: the skin circulation. Scand J Med Sci Sports. (2010) 20:29–39. doi: 10.1111/j.1600-0838.2010.01206.x 21029188

[53] Nielsen B Savard G Richter EA Hargreaves M Saltin B. Muscle blood flow and muscle metabolism during exercise and heat stress. J Appl Physiol (1985). (1990) 69:1040–6. doi: 10.1152/jappl.1990.69.3.1040 2246151

[54] Gonzalez-Alonso J Calbet JA. Reductions in systemic and skeletal muscle blood flow and oxygen delivery limit maximal aerobic capacity in humans. Circulation. (2003) 107:824–30. doi: 10.1161/01.cir.0000049746.29175.3f, PMID: 12591751

[55] Maloyan A Eli-Berchoer L Semenza GL Gerstenblith G Stern MD Horowitz M. Hif-1alpha-targeted pathways are activated by heat acclimation and contribute to acclimation-ischemic cross-tolerance in the heart. Physiol Genomics. (2005) 23:79–88. doi: 10.1152/physiolgenomics.00279.2004, PMID: 16046617

[56] Gustafsson T Puntschart A Kaijser L Jansson E Sundberg CJ. Exercise-induced expression of angiogenesis-related transcription and growth factors in human skeletal muscle. Am J Phys. (1999) 276:H679–85. doi: 10.1152/ajpheart.1999.276.2.H679, PMID: 9950871

[57] Fink WJ Costill DL Van Handel PJ. Leg muscle metabolism during exercise in the heat and cold. Eur J Appl Physiol Occup Physiol. (1975) 34:183–90. doi: 10.1007/BF00999931, PMID: 1181181

[58] Febbraio MA Snow RJ Stathis CG Hargreaves M Carey MF. Effect of heat stress on muscle energy metabolism during exercise. J Appl Physiol (1985). (1994) 77:2827–31. doi: 10.1152/jappl.1994.77.6.2827 7896628

[59] Maxwell NS Aitchison TC Nimmo MA. The effect of climatic heat stress on intermittent supramaximal running performance in humans. Exp Physiol. (1996) 81:833–45. doi: 10.1113/expphysiol.1996.sp003980, PMID: 8889481

[60] Maxwell NS Gardner F Nimmo MA. Intermittent running: muscle metabolism in the heat and effect of Hypohydration. Med Sci Sports Exerc. (1999) 31:675–83. doi: 10.1097/00005768-199905000-00009, PMID: 10331887

[61] Kuang J McGinley C Lee MJ Saner NJ Garnham A Bishop DJ. Interpretation of exercise-induced changes in human skeletal muscle Mrna expression depends on the timing of the post-exercise biopsies. PeerJ. (2022) 10:e12856. doi: 10.7717/peerj.12856, PMID: 35186464 PMC8820226

[62] Periard JD Eijsvogels TMH Daanen HAM. Exercise under heat stress: thermoregulation, hydration, performance implications, and mitigation strategies. Physiol Rev. (2021) 101:1873–979. doi: 10.1152/physrev.00038.2020, PMID: 33829868

[63] Geor RJ McCutcheon LJ Lindinger MI. Adaptations to daily exercise in hot and humid ambient conditions in trained thoroughbred horses. Equine Vet J Suppl. (1996) 22:63–8. doi: 10.1111/j.2042-3306.1996.tb05033.x, PMID: 8894552

